# Photobiomodulation (PBMT) and antimicrobial photodynamic therapy (aPDT) in oral manifestations of patients infected by Sars-CoV-2: systematic review and meta-analysis

**DOI:** 10.1186/s42269-022-00830-z

**Published:** 2022-05-16

**Authors:** Juliano Abreu Pacheco, Kelly Fernanda Molena, Camila Raíssa Oliveira Gontijo Martins, Silmara Aparecida Milori Corona, Maria Cristina Borsatto

**Affiliations:** 1 Postgraduate Program, EERP, University of São Paulo, Research Coordinator at the Ribeirão Preto Cancer Hospital, Sobeccan Hospital Foundation, Ribeirão Preto, Brazil; 2 Postgraduate Program in Pediatric Dentistry, School of Dentistry of Ribeirão Preto - University of São Paulo, Ribeirão Preto, Brazil; 3 Department of Restorative Dentistry, School of Dentistry of Ribeirão Preto, University of São Paulo, São Paulo, Brazil; 4 Department of Pediatric Dentistry, School of Dentistry of Ribeirão Preto, University of São Paulo, São Paulo, Brazil

**Keywords:** Photodynamic therapy, Low level laser therapy, LLLT, Oral manifestation, Coronavirus, Sars-CoV-2, COVID-19

## Abstract

**Background:**

In 2019, a viral and respiratory pathology called COVID-19 emerged in Wuhan, China, and spread to other continents. Its main symptoms include fever, cough, dyspnea, myalgia, anorexia and respiratory distress in the most severe cases, which can lead to death. Furthermore, manifestations in the oral cavity such as ageusia and dysgeusia, as well as lesions in other regions of the oral cavity, can be observed.

**Main body:**

This systematic review and meta-analysis aimed to critically assess the clinical evidence on the use of photobiomodulation (PBMT) and antimicrobial photodynamic therapy (aPDT) for the treatment of oral lesions in patients infected with Sars-Cov-2. The literature extracted from electronic databases such as PubMed, Medline, CINAHL, and Google Scholar was screened for eligibility, and relevant articles were included. The review is limited to manuscripts published in English, Spanish and Portuguese language between December 2019 and October 2021. A total of 5 articles with 11 cases retracting PBMT and aPDT as therapeutic strategies for the regression of oral lesions and painful symptoms. The results show favoring the associated use of PBMT with aPDT (*P* = 0.004), and the isolated use of PBMT with the result of significant “*P* = 0.005” and good confidence interval (7.18, 39.20) in ulcerative lesions, herpetic, aphthous, erythematous, petechiae and necrotic areas.

**Conclusions:**

PBMT and aPDT could be effective in the treatment of oral lesions of patients infected with Sars-Cov-2 in a short period of time; however, more long-term randomized clinical trials studies are needed to define the therapeutic strategy.

## Background

COVID-19 is an acute respiratory pathology and the set of signs and symptoms develop rapidly in a synchronous and varied manner, leading to a global characterization of the name “Severe Acute Respiratory Syndrome Coronavirus 2” (Sars-CoV-2) (Singh et al. 2021; Pacheco et al. 2021). The most common symptoms are fever, dyspnea, cough, myalgia and anorexia (Sousa and Paradella 2021; Jiang et al. 2020; Huang et al. 2020). In severe cases, pulmonary impairment occurs with increased respiratory rate (> 30 times/min.), decreased O_2_ saturation (< 93% in room air) and PaO_2_/FiO_2_ (< 300 mm Hg) are observed (Sousa and Paradella 2021; Berlin et al. 2020). Critical cases include severe acute respiratory syndrome, acute cardiac injury, and multiple organ failure (Sousa and Paradella 2021; Huang et al. 2020; Pacheco et al. 2021).

Due to this new pandemic context, and the growing expansion of the number of cases, it is possible to identify and relate a wide variety of signs and symptoms in these patients contaminated by the coronavirus with the appearance of secondary lesions in the oral cavity (Pacheco et al. 2021).

Many patients infected with Sars-CoV-2 present oral lesions, and as it is a new systemic disease, the database of these lesions is being published in case reports (Pacheco et al. 2021). However, there are reports of oral manifestations by clinicians and researchers related to various comorbidities such as ulcer, erosion, blister, vesicle, pustule, fissured or depapillary tongue, macula, papule, plaque, pigmentation, halitosis, whitish areas, hemorrhagic crust, necrosis, petechiae, swelling, erythema and spontaneous bleeding (Iranmanesh et al. 2021; Pacheco et al. 2021). The most affected sites were tongue (38%), lip mucosa (26%) and palate (22%) (Iranmanesh et al. 2021; Soares et al. 2020). Suggested diagnoses of lesions are diverse, including aphthous stomatitis, herpetic lesions, candidiasis, vasculitis, Kawasaki type, eruption by drug, periodontal necrotizing disease, bullous angina type, angular cheilitis, atypical Sweet's syndrome and Melkersson–Rosenthal syndrome (Iranmanesh et al. 2021; Pacheco et al. 2021). Oral lesions are characterized as symptomatic in 68% of cases and similar in both sexes (49% female and 51% male) (Tamang et al. 2021). And it should be noted that the lack of oral cavity hygiene, opportunistic infections, stress, immunosuppression, vasculitis and inflammatory response secondary to Sars-CoV-2 are being characterized as the most important causal factors for the appearance of these lesions in patients with Sars-CoV-2 (Tsuchiya 2021; Iranmanesh et al. 2021).

Taste disorder (dysgeusia) is the most common oral manifestation found in this niche of patients with Sars-CoV-2 (Jakubovics 2021; Pacheco et al. 2021). In mild cases, oral mucosal lesions evolve before or simultaneously with the initial respiratory symptoms; however, in patients who worsened and required medication and hospitalization, oral lesions had an evolution process approximately between 7 and 24 days after the onset of symptoms (Dos Santos et al. 2021; Pacheco et al. 2021).

There are physiological structures in the oral cavity available through cell receptors called Angiotensin-Converting Enzyme 2 (ACE2) (Jia et al. 2005). The coronavirus has in its structure the glycoprotein S, which is the key factor for the entry of Sars-CoV-2 into the cell, which contains two functional domains: S1 (Zhong et al. 2020) which is an angiotensin converting enzyme receptor binding domain 2 (ACE2) and the S2 which is required for coronavirus fusion and cell membranes. There is a resonance in global studies that ACE2 is likely the receptor for Sars-CoV-2 (Zhong et al. 2020; Cassol-Spanemberg et al. 2016) which could induce an increase in the susceptibility and/or progression of COVID-19 disease, and this severity would be closely linked to increased viral load in the oropharyngeal region with eventual systemic repercussions for respiratory epithelial cells (Zhong et al. 2020).

In case of minimizing these secondary manifestations caused by the Sars-CoV-2 infection, low intensity laser therapy (LLLT) emerges as a practical tool and is characterized by its non-invasiveness to biological tissues, which through a wavelength (red or infrared) stimulates responses to injured tissue by promoting anti-inflammatory, analgesic and healing effects. LLLT promotes the effect of photobiomodulation (PBMT) which is able to regulate antioxidant defenses and reduce oxidative stress (Cassol-Spanemberg et al. 2016).

PBMT is based on the interaction of light with biological tissues, stimulating photo-physical, chemical and biological events in the cell, in the search for better therapeutic responses, consequently altering cell metabolism. It is known that this therapy can currently bio-stimulate or bio-inhibit cells depending on different variables relevant to light (Cassol-Spanemberg et al. 2016). The therapeutic effects of low-power laser irradiation have been investigated in several health areas (Bensadoun et al. 2020). Some beneficial effects such as improved immunosuppression-immunostimulation, stabilization of autoimmune disease and nerve regeneration have been reported in the literature (Zecha et al. 2016; Cassol-Spanemberg et al. 2016).

The basic physiological effects and mechanisms of PBMT are widely discussed in the world literature and have excellent results in healing processes, decontamination, biomodulation of the inflammatory process, and pain control. Indications for clinical applications of PBMT at the cellular level are broad within the healthcare field, and include interdisciplinary therapies that involve the treatment of different pathologies, as well as pain control in general (Pacheco et al. 2021).

It should be noted that the imbalance of the oral cavity microbiota may provide the functional instability of the oropharynx with the development of numerous secondary pathogens related to systemic diseases. This instability open space for colonization by microorganisms in other organs that may evolve into an undesirable effect as they produce potent toxic agents (Pacheco et al. 2021). The oral microbiome in patients has a greater bacterial sensitivity and specificity due to the vulnerability of the immune system. In this context, PBMT proposes another interesting aspect for the decontamination of the oral microbiota in these patients, given that the biosensors contained in the oral region through photodynamic therapy will activate a photosensitizing substance by a red laser with a high efficiency in the decontamination of microorganisms, such as bacteria, viruses and fungi. We call this process antimicrobial photodynamic therapy (aPDT) (Carrera et al. 2016; Pacheco et al. 2021).

The aPDT has photosensitization as a mechanism, which consists of the direct action of light with a photosensitizing agent together with oxygen. This process induces the production of free radicals that promote microbial lysis (Pacheco et al. 2021), promoting decontamination (Issa and Manela-Azulay 2010). This therapy is of low cost, basically without side effects, and the possibility of controlling the oral microbiota (Pacheco et al. 2021).

When a photosensitizing substance, such as methylene blue, for example, is photoactivated in the presence of oxygen, it leads to the generation of reactive oxygen species (Vittar et al. 2008), causing irreversible damage to target structures such as proteins and lipids, leading to death of microorganisms by apoptosis or necrosis (Conrado et al. 2021; Castano et al. 2005). In viral infectious agents, aPDT can affect the lipid or protein structures of the viral envelope membrane, even the nucleic acid, regardless of the specific interaction with a receptor, indicating that it is an efficient tool to eliminate these infectious agents (Conrado et al. 2021; Castano et al. 2005; Wiehe et al. 2019; Monjo et al. 2018; Pacheco et al. 2021).

The aim of this manuscript is to present a systematic review of the literature on the use of PBMT and aPDT in oral lesions of patients affected by the Sars-CoV-2 infection, reviewing all relevant observational studies to answer the following question: photobiomodulation (PBMT) and antimicrobial photodynamic therapy (aPDT) are effective to treat oral manifestations of patients infected with Sars-CoV-2?

## Methods

The methodology was defined following the PRISMA guidelines (Preferred Systematic Reviews and Meta-Analysis Report) (Moher et al. 2015), and registered in the database in the International Prospective Registry of Systematic Reviews (PROSPERO) under registration CRD42021250106.

The literature search was conducted in 4 databases and in gray literature, whose question focused in the review was “Photobiomodulation (PBMT) and antimicrobial Photodynamic Therapy (aPDT) are effective to treat oral manifestations of patients infected by Sars-CoV-2?”. The flow diagram was used as the strategy design in this study.

### Literature research

A search was performed in the PubMed, Medline, CINAHL, Google Scholar databases, as well as a manual search of the reference lists of the included studies, from January 1, 2019, to October 16, 2021. The first phase established an investigation for define MeSH (Medical Subject Headings) terms to ensure high sensitivity and accuracy, and researchers tracked abstract and publication titles (Conrado et al. 2021). The MESH terms used in searches across all databases were: “COVID 19” OR “Sars-Cov-2” OR “Coronavirus infections” OR coronavirus AND “Photodynamic therapies” OR “Photodynamic therapy” OR “PDT methylene blue” OR “Cold laser decontamination” OR “Photodynamic LLLT” OR Photobiomodulation AND “Oral manifestations” OR “Oral injury” AND “Angiotensin II” OR “ACE2”.

### Election criteria

Articles describing photobiomodulation therapy and antimicrobial photodynamic therapy as treatment for oral manifestations in patients infected by the Sars-CoV-2 infection, based on titles and abstract, were included in this review (Conrado et al. 2021). Original articles in English, Spanish and Portuguese within the defined period (2019–2021) with abstract available in the database were evaluated and validated within the inclusion criteria. Inclusion criteria were defined using the PICO strategy (Population, Intervention, Comparison and Outcome) (Table 1) and consisted of "in vivo" studies and case reports, excluding works published as letters to the editor, systematic reviews and articles not available in full.

**Table 1:** PICO Strategy with inclusion criteria using on research

PICO strategy	
Population	Patients infected by the Sars-CoV-2 infection who had oral lesions and were treated with PBMT or aPDT laser therapy
Intervention	Patients were exposed to photobiomodulation therapy (PBMT) and/or antimicrobial photodynamic therapy (aPDT)
Comparison	Comparison between patients who did not use PBMT and/or aPDT
Outcome	Observe whether or not there was improvement in the lesions after using PBMT and aPDT

### Selection and quality assessment of relevant studies

Articles selected according to the selection criteria were retrieved in PDF format, numbered and randomly distributed among three researchers. The reference list was manually checked by the researchers in order to retrieve publications that were not previously found in the database search, to increase the sensitivity and quality of the review (Contado et al. 2021). A consensus meeting was held to discuss divergences after evaluating the quality of publications and guaranteeing the validation of the articles selected by the researchers.

### Data extraction

Articles included in the study were randomly distributed among researchers to collect relevant data (Conrado et al. 2021).

Through a table, important data were selected, including: author and year of publication, local and type of lesion, applied technique and parameter of laser and outcome, whether there was improvement of the lesion, in how many days there was improvement, whether the type of light source interfered and to observe whether these complementary therapies would be effective or not in the treatment of oral lesions in patients affected by Sars-CoV-2. With the results collected, the data were evaluated one by one, each case addressed in the literature was presented in each row of the table.

### Bias risk

Quality and risk of bias in selected articles were independently performed by two authors, using the Joanna Briggs Institute's Critical Assessment Checklist for systematic reviews and research summaries (Moola et al. 2015). The third author was consulted in case of disagreement. Scoring decisions were discussed by all reviewers prior to assessments. Checklists allow for the quantification of scores, where high scores represent a low risk of bias; low scores indicate a high risk of bias or "unclear" domains in publications (Conrado et al. 2021).

## Results

### Selection and heterogeneity of studies

**Fig. 1:**
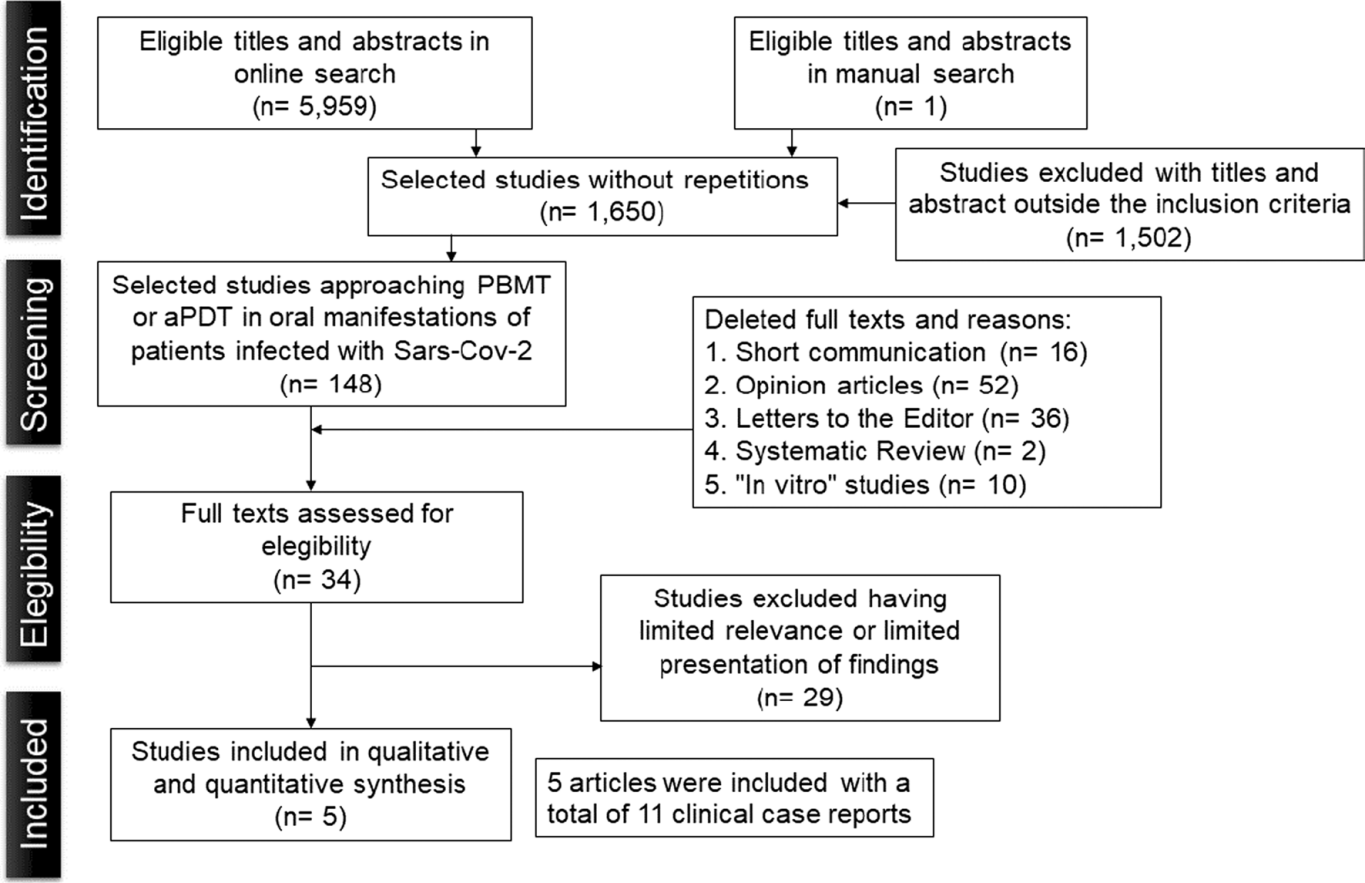
Literature search flow diagram and selection criteria adapted from PRISMA (Preferred report items for systematic reviews and meta-analysis)

In the first phase, 5,959 articles were identified in the databases and, after removing the duplicates, 1,650 articles remained for reading the title and abstract. After evaluating all records, 32 articles were selected where they were read in full and following the inclusion and exclusion criteria, 5 articles were finally included, as instructed in the Infographic (Fig. 1).

All studies were carried out in Brazil; they were case reports in the literature and were published between January and October 2021, in English, where patients were admitted to hospitals with symptoms characteristic of COVID-19 and later confirmed through the examination RT-PCR. However, they used different therapeutic approaches for lesions in the oral cavity, including PBMT (Brandão et al. 2021; Baeder et al. 2021), PBMT associated with aPDT (Teixeira et al. 2021; Ramires et al. 2021; Garcez et al. 2021) and awaiting spontaneous regression of the lesions without any laser intervention (Brandão et al. 2021; Baeder et al. 2021). Thus, 5 articles found in the literature presented a total of 11 cases using PBMT and aPDT as therapeutic strategies for the regression of oral lesions and painful symptoms in patients with COVID-19 (Table 2).

**Table 2:** Characteristics of the included studies, according to Author and year of publication; Place and type of injury, Technique and Parameters in the use of Laser and outcome

Author and Year	Local and type of lesion	Technique applied and Laser parameter	Outcome
Brandão et. al. (2021)	Upper lip, lower lip and anterior dorsum of tongue. Necrotic and aphthous ulcers	PBMT. 40 mW, beam area 0.04 cm^2^, 1 W/cm 2 irradiance, energy 0.4 J and 10 J/cm^2^ creep and 660 nm	Symptom relief within 2 days and complete resolution of lesions within 11 days
Brandão et. al. (2021)	Upper lip, lower lip and anterior dorsum of tongue. Necrotic areas and aphthous ulcers	PBMT. 40 mW, beam area 0.04 cm^2^, 1 W/cm^2^ irradiance, energy 0.4 J and 10 J/cm^2^ creep and 660 nm	Improvement of intraoral lesions in 10 days and lip ulcerations there was no improvement until the publication of the case, as well as the clinical case of the patient
Brandão et. al. (2021)	Lateral edge of tongue and palate. Petechiae and necrotic areas	PBMT. 40 mW, beam area 0.04 cm^2^, 1 W/cm 2 irradiance, energy 0.4 J and 10 J/cm^2^ creep and 660 nm	Total pain control in 5 days
Brandão et. al. (2021)	Upper and lower lip mucosa. Necrotic and hemorrhagic ulcers	PBMT. 40 mW, beam area 0.04 cm^2^, 1 W/cm 2 irradiance, energy 0.4 J and 10 J/cm^2^ creep and 660 nm	Pain regression and clinical improvement in 7 days
Teixeira et. al. (2021)	Upper and lower lip injury. Hemorrhagic and necrotic ulcers	PBMT and aPDT. 100 mW, 33 J/cm^2^, 0.5 J and 5 s per point. A total of 6 points were distributed for the injuries. Soon after, an aPDT technique was performed, with 0.01% methylene blue applied to all lesions, and after 3 min (pre-irradiation time), the same laser parameters were used, but providing 40 s (4 J) per lesion, 660 nm	Total improvement of the lesion in 3 days and healing within the first 24 h
Teixeira et. al. (2021)	Upper and lower lip injury. Erythematous lesions	PBMT and aPDT. 100 mW, 33 J/cm^2^, 0.5 J and 5 s per point. A total of 6 points were distributed for the injuries. Soon after, an aPDT technique was performed, with 0.01% methylene blue applied to all lesions, and after 3 min (pre-irradiation time), the same laser parameters were used, but providing 40 s (4 J) per lesion, 660 nm	Total improvement of the lesion after 24 h
Teixeira et. al. (2021)	Upper and lower lip injury. Painful scaly lip lesions	PBMT and aPDT. 100 mW, 33 J/cm^2^, 0.5 J and 5 s per point. A total of 6 points were distributed for the injuries. Soon after, an aPDT technique was performed, with 0.01% methylene blue applied to all lesions, and after 3 min (pre-irradiation time), the same laser parameters were used, but providing 40 s (4 J) per lesion, 660 nm	Total improvement of lesions in 3 days
Teixeira et. al. (2021)	Upper and lower lip injury. Painful scaly lip lesions	PBMT and aPDT. 100 mW, 33 J/cm^2^, 0.5 J and 5 s per point. A total of 6 points were distributed for the injuries. Soon after, an aPDT technique was performed, with 0.01% methylene blue applied to all lesions, and after 3 min (pre-irradiation time), the same laser parameters were used, but providing 40 s (4 J) per lesion, 660 nm	Complete resolution of lesions within 4 days
Ramires et. al. (2021)	Upper and lower lip injury. Extensive necrotic ulcers	PBMT and aPDT. aPDT was performed for 2 days. For this, 0.01% methylene blue was applied to all lesions after 5 min (pre-irradiation time), 100 mW, 32.14 J/cm^2^, 9 J and 9 s per point and PBMT 100 mW, 17.8 J/cm^2^, 1 J and 10 s of irradiation per spot at 660 and 808 nm, using a laser device programming tool that changes the wavelength periodically (every 5 s)	Wound healing in 4 days
Baeder et. al. (2021)	Region between attached gingiva and palate. Ulcers, erythema and vesicles	PBMT. 3 J every 2 days for 1 week, 660 nm	Burning ceased in 7 days and after 14 days, the lesions disappeared completely
Garcez et. al. (2021)	Upper and lower lips and inner labial mucosa of the gingiva. Edema with mucosal desquamation, ulceration and blood crusts on the inner surface of the labial mucosa, gingival petechiae and erythematous/pseudomembranous lesions on the dorsum of the tongue, suggestive of candidiasis	PBMT and aPDT. aPDT was performed using a low power laser and methylene blue as a photosensitizer. 300 μM aqueous methylene blue solution was applied to the lips, palate and tongue with a cotton swab for 1 min, followed by irradiation of a laser light source operating at 100 mW and 660 nm with the following protocol: 90 s, resulting in an energy of 9 J per irradiation point and an energy density of 300 J/cm^2^, total of 6 points—including lips (4 points), palate (2 points) and tongue (4 points), the total irradiation time was 15 min. After the aPDT sessions, oral lesions were irradiated with 2 J of energy per point to cover the oral mucosa surface bilaterally (5 points on each side) using the same equipment, resulting in 20 s of irradiation per point and energy density per stitch of 66 J/cm^2^	Oral lesions improved after 3 days of aPDT after which treatment was followed with PBM for 4 days. The patient did not complain of discomfort in the tongue and lips after starting the photodynamic treatment with an antimicrobial

Of these, 3 were male (27%) and 8 female (73%), where 8 patients were aged over 60 years (mean 75 years) and 3 were younger than 60 years (mean 47 years).

The place most affected by the lesions were upper and lower lip (33.3%), followed by tongue (12.5%), hard palate (8.3%), buccal mucosa (4.1%) and attached gingiva (4,1%).

Among the most frequent types of lesions are ulcers (50%), herpetic lesions (40%), in addition to the rest being expressed by necrotic, erythematous, vesicular and petechiae areas.

The risk of bias assessment was established using the Joanna Briggs Institute (Moola et al. 2015) Critical Assessment List for case series, and the articles analyzed here had a low risk of bias, between 20 and 40% (Table 3).

**Table 3:** Risk of bias assessed by the Joanna Briggs Institute Critical appraisal checklist for case series

Authors	Q.1	Q.2	Q.3	Q.4	Q.5	Q.6	Q.7	Q.8	Q.9	Q.10	%yes/risk
Baeder et al. (2021)	U	√	√	√	√	√	√	√	–	N/A	70%/30%
Brandão et al. (2021)	U	√	√	√	√	√	√	√	–	N/A	70%/30%
Ramires et al. (2021)	√	√	√	N/A	N/A	√	√	√	–	N/A	60%/40%
Teixeira et al. (2021)	√	√	√	√	√	√	√	√	–	N/A	80%/20%
Garcez et al. (2021)	√	√	√	N/A	√	√	√	√	–	N/A	70%/30%

√-yes; –-No; U-Unclear; N/A-not applicable

### Photosensitizer and light sources

Brandão et al. (2021), after not observing improvement in vesicular, erythematous and vesicle lesions with the use of antiviral therapy with intravenous Acyclovir (250 mg), 3 times a day for 7 days, used PBMT (Twin Flex, MMOptics, São Carlos, Brazil) with the therapeutic protocol for oral mucositis associated with cancer therapy, in patients with COVID-19, where the laser device was positioned perpendicular to the surface of the oral ulcers, for 10 s per site, operating at a wavelength of 660 nm, 40 mW average power, 0.04 cm^2^ beam area, 1 W/cm^2^ irradiance, 0.4 J energy and 10 J/cm^2^ creep.

Baeder et al. (2021) proposed the treatment of herpetic lesions that did not spontaneously regress in patients infected with COVID-19 through the use of PBMT with a wavelength of 660 nm, with a dose of 3 J every 48 h, in addition to the mouthwash with 0.12% chlorhexidine twice daily for 7 days.

Teixeira et al. (2021) and Ramires et al. (2021) used similar protocols, the first one using an association of aPDT and PBMT for two days. For PBMT, a DUO® Laser device (MMOptics, São Carlos, SP, Brazil) was used at 660 nm, contact mode, point to point, with 100 mW, 33 J/cm^2^, 0.5 J and 5 s per point. A total of 6 points were distributed for necrotic lesions, bleeding ulcers and erythematous lesions in patients with COVID-19. Soon after, an aPDT technique was performed, with 0.01% methylene blue applied to all lesions, and after 3 min (pre-irradiation time), the same laser parameters were used, but providing 40 s (4 J) for injury. Ramires et al. (2021) differed in the pre-irradiation time, which waited 5 min, and the laser device used was the Therapy EC® (DMC, São Carlos, SP, Brazil) at 660 nm, contact mode, point to point, with 100 mW, 32.14 J/cm^2^, 9 J and 9 s per stitch. In this case, a total of 30 points were distributed over the affected areas: 20 points on the upper lip and 10 on the lower lip. In addition, on the second day, a PBMT session was held. The same areas described above were irradiated with the same equipment, but using 100 mW, 17.8 J/cm^2^, 1 J and 10 s of irradiation per point at 660 and 808 nm, using a programming tool that triggers the two lengths simultaneously (every 5 s).

Garcez et al. (2021) used a protocol of 3 sessions of aPDT (660 nm diode laser + methylene blue) on the lips and tongue, every 24 h to control contamination, followed by PBMT (low power laser, 100 mW, 2 J/point) for the lips, tongue and oral mucosa for four additional sessions every 24 h, in ulcerative lesions, hemorrhagic crusts, petechiae and erythematous lesions in patients with COVID-19.

### Synthesis of meta-analysis

The synthesis of statistical data was performed through meta-analysis, which consists of the statistical combination of results from two or more separate studies. Potential advantages of meta-analysis include an improvement in accuracy, the ability to answer questions not posed by individual studies, and the opportunity to resolve disputes arising from conflicting claims (Deeks et al. 2021). Meta-analysis was performed on groups of studies that were homogeneous in terms of similarity in population, interventions, and outcomes to provide a meaningful summary (Higgins et al. 2021). Most of the scientific articles selected in this study presented a low risk of bias and corroborated the final production of the analysis.

It should be noted that the results of the 11 clinical cases referring to the 5 articles of the study were evaluated through meta-analysis, considering that these studies presented homogeneous data quantitatively evaluating the use of PBMT and aPDT for the reduction of oral lesions in patients affected by COVID-19.

### Repair of oral lesion by PBMT and aPDT/PBMT

**Fig. 2:**
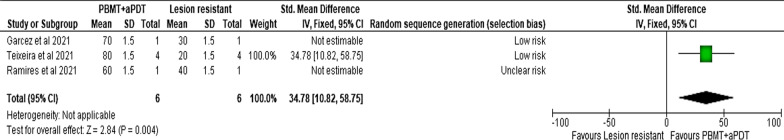
Forest plot demonstrating meta-analysis for oral manifestations for COVID-19 patients facing the treatment of PBMT associated with aPDT

The results show favoring the associated use of PBMT with aPDT (*P* = 0.004) (Pacheco et al. 2021), considering that there was a greater reduction in ulcerative, erythematous, necrotic and petechiae oral lesions in these patients positive by COVID-19, which is demonstrated in the “Forest plot” confidence interval (Fig. 2). The fixed model was applied in this meta-analysis and heterogeneity is not a considerable factor for the analysis.

Heterogeneity was analyzed through the analysis of the approximate guide for interpretation in the context of meta-analysis of randomized clinical trials, which is made available by the Cochrane Group of Statistical Methods (Higgins et al. 2021), according to the source of the Review Manager 5.4.1 program and Deeks et al. (2021); as described below:

- 0% to 40%: may not be important;
- 30% to 60%: may represent moderate heterogeneity;
- 50% to 90%: may represent substantial heterogeneity;
- 75% to 100%: considerable heterogeneity.

**Fig. 3:**
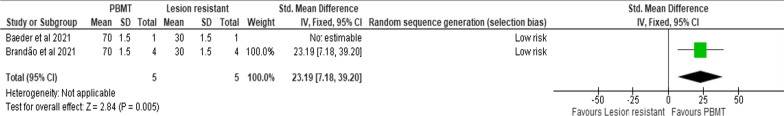
Forest plot demonstrating meta-analysis for oral manifestations for COVID-19 patients facing PBMT treatment

The isolated use of PBMT, according to the meta-analysis, was also effective in the treatment of oral lesions caused by COVID-19 (Santos et al. 2021), with the result of a significant “*P* = 0.005” and a good confidence interval (7.18, 39.20) (Pacheco et al. 2021) (Fig. 3).

### Analysis of the main results

PBMT without association with other laser therapy (Brandão et al. 2021; Baeder et al. 2021), the relief of painful symptoms occurred between 2 and 14 days with total healing of the lesion, excluding one case (Case 2) (Brandão et al. 2021), where until the date of publication of the article there was no response to laser therapy or regression of the COVID-19 infection picture.

PBMT associated with aPDT showed the best results with relief of painful symptoms in the first 24 h and healing of the lesion within 4 days (Teixeira et al. 2021; Ramires et al. 2021; Garcez et al. 2021).

Cases where laser therapy was not used as adjuvant, were young patients, mean age of 38.2 years and the lesions had their spontaneous regression within a considerable time, between 4 and 16 days.

### Effects of PBMT and aPDT on Sars-CoV-2 contaminated oral tissues

PBMT allowed relief of painful symptoms and complete resolution of lesions in the clinical cases presented in this review. When associated with aPDT, this improvement occurred in a shorter time span, thus suggesting that it is an effective therapy to treat oral lesions in patients affected by COVID-19 (Teixeira et al. 2021).

## Discussion

The pandemic process related to COVID-19 in the last two years, current studies related to morbid changes in the oropharyngeal region are in the process of evolution, as therapies for the repair of secondary lesions promoted by Sars-CoV-2 are ongoing. In an incipient way in the health-disease process, as well as the impossibility of clinicians and patients to evaluate a reduced amount of case studies (Pacheco et al. 2021) or long-term research.

In this view, systematic reviews aim to ensure that clinical decisions are made with an up-to-date and complete understanding of the relevant scientific evidence (Lasserson et al. 2021). Judiciously evaluating the scientific literature, systematic reviews provide an up-to-date, qualitative summary of the state of research knowledge about an intervention, address the main research problem, namely, that of bias; as well as direct new research projects, indicating specific gaps in knowledge or whether evidence is lacking (Chalmers et al. 2014; Lasserson et al. 2021).

The present study verified in the available scientific literature whether there was evidence regarding the efficacy and safety of the clinical applicability of PBMT and aPDT as effective options to treat oral manifestations of patients infected by COVID-19 (Chalmers et al. 2014; Pacheco et al. 2021), through a review of narrative case reports from current literature. The studies included in this research evaluated 5 scientific articles, where 11 clinical cases had similar interventions. All clinical cases evaluated in this systematic review demonstrated positive therapeutic effects that promoted a global impact on patients' health.

The focus of the systematic review was to address the use of PBMT, and/or aPDT/PBMT in the cytotoxic reduction of secondary manifestations promoted by COVID-19 (Pacheco et al. 2021; Conrado et al. 2021) that manifest themselves in the oral mucosa, through ulcers, erosions, aphthous lesions, plaques, hemorrhagic crusts among other alterations, being the tongue, labial mucosa and palate the most affected places (Iranmanesh et al. 2021).

Lesions of the stomatognathic system that evolve negatively during the therapeutic process have a refractory potential to compromise and exacerbate immunosuppression, which adds to the appearance of opportunistic infections arising from atypical changes in oral mucosa cells, such as aphthous stomatitis (Pacheco et al. 2021), herpetic lesions and candidiasis.

It is extensive in the current literature that the main receptor for Sars-CoV-2 is ACE2, which would be closely linked to the imbalance of the oral microbiome that could induce an increase and sharp progression of the viral load in the oropharyngeal region with an eventual undesirable systemic repercussion (Zhong et al. 2020).

Furthermore, PBMT and aPDT could become associative or complementary therapeutic strategies, as they present themselves as an auxiliary tool with a positive multi and interdisciplinary nature in the focal control of pathologies of the oropharyngeal tract, in addition to being a low-cost, painless and non-invasive technique (Hennessy and Hamblin 2017). Furthermore, techniques related to low intensity laser therapy constitute, in the contemporary context, a form of treatment of painful symptoms and clinical picture of these diseases in the oral cavity by stimulating analgesic, anti-inflammatory and anti-edematous effects (Zecha et al. 2016; Carrera et al. 2016; Castano et al. 2005; Wiehe et al. 2019; Monjo et al. 2018).

This systematic review contributes to the amount of 11 clinical cases that used PBMT and/or aPDT as therapeutic resources for lesions in the oral cavity of patients affected by COVID-19, or awaiting natural regression of the lesions with topical medication in some cases (Brandão et al. 2021; Baeder et al. 2021; Teixeira et al. 2021; Ramires et al. 2021). The cases were patients admitted to public and private hospitals with symptoms characteristic of COVID-19 and confirmed through the RT-PCR (Corman et al. 2020). Most were female (73%), where 8 patients were aged over 60 years (average 75 years old) and 3 were younger than 60 years old (average 47 years old). The studies were conducted in Brazil, which creates the need for manuscripts in other regions of the world.

In the observational criterion for the incidental evaluation of oral lesions, in ascending order of nature of these manifestations, there was an involvement of ulcerative lesions, necrotic areas, herpetic lesions, erythematous and petechiae (Iranmanesh et al. 2021). Regarding the nature of the oral lesion, in ascending order: upper and lower lips, tongue, hard palate, buccal mucosa and attached gums, which is in agreement with the findings in the literature (Soares et al. 2020; Iranmanesh et al. 2021; Amorim Dos Santos et al 2021).

Brandão et al. (2021) and Baeder et al. (2021) using protocols pertinent to the case, did not observe apparent regression of herpetic lesions on the lip, and used PBMT with clinical improvement of the lesions total tissue repair between 5 and 14 days. When they awaited spontaneous regression of the lesions, medications such as Acyclovir, mouthwash with 0.12% chlorhexidine, ascorbic acid (Brandão et al. 2021; Baeder et al. 2021), were used, in addition to individual medication protocols for each patient due to COVID-19 infection (Lofti et al. 2020; Carlotti et al. 2020; Pacheco et al. 2021).

Teixeira et al. (2021), Ramires et al. (2021) and Garcez et al. (2021) implemented associative protocols between the PBMT and aPDT techniques, and observed relief of painful symptoms in the first 24 h and healing of the lesion within 4 days, in accordance with the findings of Maya et al. (2020) for the treatment of palatal ulcers.

In the observational evaluation criteria of the 5 articles in question, it is important to emphasize that oral lesions appeared in these patients positive by COVID-19 (Cruz Tapia et al. 2020), concomitantly with loss of taste and smell (Mutiawati et al. 2021), worsening of COVID-19 symptoms and were more severe in people older and systemically compromised (Brandão et al. 2021).

Another considerable score is that these lesions were characterized by necrotic ulcers and with great painful symptoms. In young patients, with mild COVID-19, they presented as aphthous ulcers.

The risks of bias were analyzed following the Joanna Briggs Institute's Critical Assessment Checklist for systematic reviews and research summaries (Moola et al. 2015), and was between 60 and 80% in the studies, that is, the closer to 100%, the more reliable the model contextualizes the variability of the response data around the mean, and the risk of bias between 20 and 40% and the lower this risk, the greater the methodological quality (Pacheco et al. 2021).

And it is worth noting the inclusion of the meta-analysis in this systematic review that highlights the intervention with PBMT and aPDT/PBMT with evidence of efficacy corresponding to the 5 studies included, as well as the action of the therapeutic theme in the reduction of secondary lesions in the oropharyngeal region caused by COVID-19, who showed a statistically significant improvement in tissue repair and consequent quality of life. This also alerts about the importance of the dental surgeon in an intensive care unit, as part of the multi-disciplinary team, for the recovery of patients infected with COVID-19, in the improvement of the clinical and symptomatological condition (Amorim et al. 2020). However, it is noteworthy to report that the small number of cases in the literature opens the need for the publication of more articles using these therapeutic protocols, especially including randomized clinical trials.

Therefore, a total of 11 cases were presented here; in this way, we can suggest that PBMT and aPDT could be effective therapies, both isolated, but mainly when associated, as they are relatively low-cost therapies, of practical employability in hospital or outpatient settings, and that they proved to be effective in repairing the clinical picture of oral lesions of different nature and locations, in these patients affected by COVID-19 in a short period of time. However, as it is a recent disease on the world stage and non-random publications, it is necessary that further research on the benefits and harms of the use of low-intensity laser is carried out so that it is really effective in reducing and neutralization of diseases of the oropharyngeal tract in detriment of the probable high viral load of Sars-CoV-2 (Pacheco et al. 2021), for example, investigating the immunoexpression of ACE2 in oral lesions or saliva, and evaluating the possibility of these therapies inhibiting or altering this receptor at these sites.

Therefore, based on current findings, we suggest conducting new randomized clinical trials, which contain a well-developed methodology and include case descriptions with specific protocols for each lesion, in order to enrich the findings in these patients, and also define therapeutic protocols concrete for patients with COVID-19 who present lesions in the oral cavity (Sousa and Paradella 2021).

## Conclusions

Based on the findings of this systematic review and literature meta-analysis, PBMT and aPDT were effective in the treatment of oral lesions in patients infected with COVID-19 in a short period of time.

The simultaneous use of PBMT/aPDT techniques, with different wavelengths, and the use of the photosensitive substance of methylene blue enhance the decontamination action of the oral cavity with eventual repair of the affected cells.

It is important to emphasize how meager the results' literature analysis were and that based on current literature no recommendation can be given, except that properly controlled long-term studies are needed with enough patient material.

## Data Availability

Not applicable.

## Abbreviations

PBMT: Photobiomodulation
aPDT: Antimicrobial photodynamic therapy
Sars-CoV-2: Severe acute respiratory syndrome Coronavirus 2
PRISMA: Preferred report on systematic reviews and meta-analysis
PROSPERO: International prospective registry of systematic reviews
LLLT: Low intensity laser therapy
COVID-19: Coronavirus disease 2019
ACE2: Angiotensin-converting enzyme 2
MeSH: Medical subject headings
PICO: Population, intervention, comparison, outcome
RT-PCR: Reverse transcription polymerase chain reaction
